# Developing Cut-off Values for Low and Very Low Bone Mineral Density at the Thoracic Spine Using Quantitative Computed Tomography

**DOI:** 10.1007/s00223-024-01268-3

**Published:** 2024-08-16

**Authors:** Andia Cheneymann, Josephine Therkildsen, Laust Dupont Rasmussen, Jesper Thygesen, Christin Isaksen, Ellen-Margrethe Hauge, Simon Winther, Morten Böttcher

**Affiliations:** 1 Department of Cardiology, University Clinic for Cardiovascular Research, Gødstrup Hospital, Hospitalsparken 15, 7400 Herning, Denmark; 2 Department of Rheumatology, Aarhus University Hospital, Palle Juul-Jensens Blvd. 99, Aarhus, Denmark; 3 Department of Clinical Medicine, Aarhus University, Palle Juul-Jensens Blvd. 11, Aarhus, Denmark; 4 Department of Cardiology, Aalborg University Hospital, Hobrovej 18-22, Aalborg, Denmark; 5 Department of Clinical Engineering, Aarhus University Hospital, Aarhus, Denmark; 6 Department of Radiology, Silkeborg Hospital, Falkevej 1D, Silkeborg, Denmark

**Keywords:** Osteoporosis, Screening, Computed tomography, Lumbar spine, Thoracic spine, Bone mineral density

## Abstract

Osteoporosis is under-diagnosed while detectable by measuring bone mineral density (BMD) using quantitative computer tomography (QCT). Opportunistic screening for low BMD has previously been suggested using lumbar QCT. However, thoracic QCT also possesses this potential to develop upper and lower cut-off values for low thoracic BMD, corresponding to the current cut-offs for lumbar BMD. In participants referred with chest pain, lumbar and thoracic BMD were measured using non-contrast lumbar- and cardiac CT scans. Lumbar BMD cut-off values for very low (< 80 mg/cm^3^), low (80–120 mg/cm^3^), and normal BMD (> 120 mg/cm^3^) were used to assess the corresponding thoracic values. A linear regression enabled identification of new diagnostic thoracic BMD cut-off values. The 177 participants (mean age 61 [range 31–74] years, 51% women) had a lumbar BMD of 121.6 mg/cm^3^ (95% CI 115.9–127.3) and a thoracic BMD of 137.0 mg/cm^3^ (95% CI: 131.5–142.5)*, p* < *0.001*. Categorization of lumbar BMD revealed 14%, 35%, and 45% in each BMD category. When applied for the thoracic BMD measurements, 25% of participants were reclassified into a lower group. Linear regression predicted a relationship of Thoracic BMD = 0.85 * Lumbar BMD + 33.5, yielding adjusted thoracic cut-off values of < 102 and > 136 mg/cm^3^. Significant differences in BMD between lumbar and thoracic regions were found, but a linear relationship enabled the development of thoracic upper and lower cut-off values for low BMD in the thoracic spine. As Thoracic CT scans are frequent, these findings will strengthen the utilization of CT images for opportunistic detection of osteoporosis.

**Supplementary Information:**

The online version contains supplementary material available at 10.1007/s00223-024-01268-3.

## Introduction

Osteoporosis is an under-diagnosed and treatable disease [1]. Annually, osteoporosis causes more than 9 million fractures globally, which increases morbidity and reduces survival worldwide [2]. Relevant interventions including medical therapy can potentially prevent fractures in nine out of ten patients [3]. These facts suggests that current screening guidelines are inadequate.

Bone mineral density (BMD) is a surrogate marker of osteoporosis. Guidelines recommend BMD testing for osteoporosis using dual-energy X-ray absorptiometry (DXA) of the hip and/or lumbar spine [4], while this modality has not been feasible for measuring thoracic spine BMD due to various reasons, including the susceptibility of the overlying sternum and ribs [5]. In general, DXA is susceptible to measurement errors from calcified tissue and degenerative spine diseases, which both are prevalent findings in individuals with high age, as it measures the tissue in two-dimensions (mg/cm^2^), entailing both the trabecular and cortical bone compartment [5].

Quantitative computed tomography (QCT) can measure BMD from CT scans with high reproducibility [6, 7]. By measuring a three-dimensional volumetric BMD (in mg/cm^3^), QCT limits measurement errors by assessing the trabecular bone compartment [8]. Consequently, QCT may result in higher precision for predicting fractures compared to DXA [9]. Additionally, QCT enables opportunistic screening of osteoporosis without additional imaging, radiation exposure, or patient time needed, as CT scans performed on various clinical indications can be used [10, 11].

Currently, the utilization of QCT to diagnose osteoporosis is based on a measurement of the hip, yielding a *T*-score like the hip or lumbar spine *T*-score assigned by DXA [12]. The *T*-score can be used to diagnose osteoporosis if the score is < –2.5 fractions of the normal population distribution of BMD [13]. The American College of Radiology (ACR) have published BMD categories for QCT of the lumbar spine, with BMD cut-off values set to approximate the *T*-score fractions of the established QCT hip measurement [14]. In general, lumbar and thoracic BMD are highly correlated [15], but BMD increases cranially [16, 17]. A previous study reports very low thoracic BMD to be prevalent among patients undergoing routine cardiac CT [18] and to be associated with an increased fracture rate [19]. However, very low thoracic BMD has not been used for diagnosing osteoporosis, and thoracic BMD cut-off values have not been developed nor compared to the current ACR-recommended lumbar cut-off values.

Thus, we aimed to investigate the correlation and differences between lumbar and thoracic spine BMD and to develop BMD cut-off values for the thoracic spine.

## Materials and Method

### Study Design and Participant Selection

**Fig. 1:**
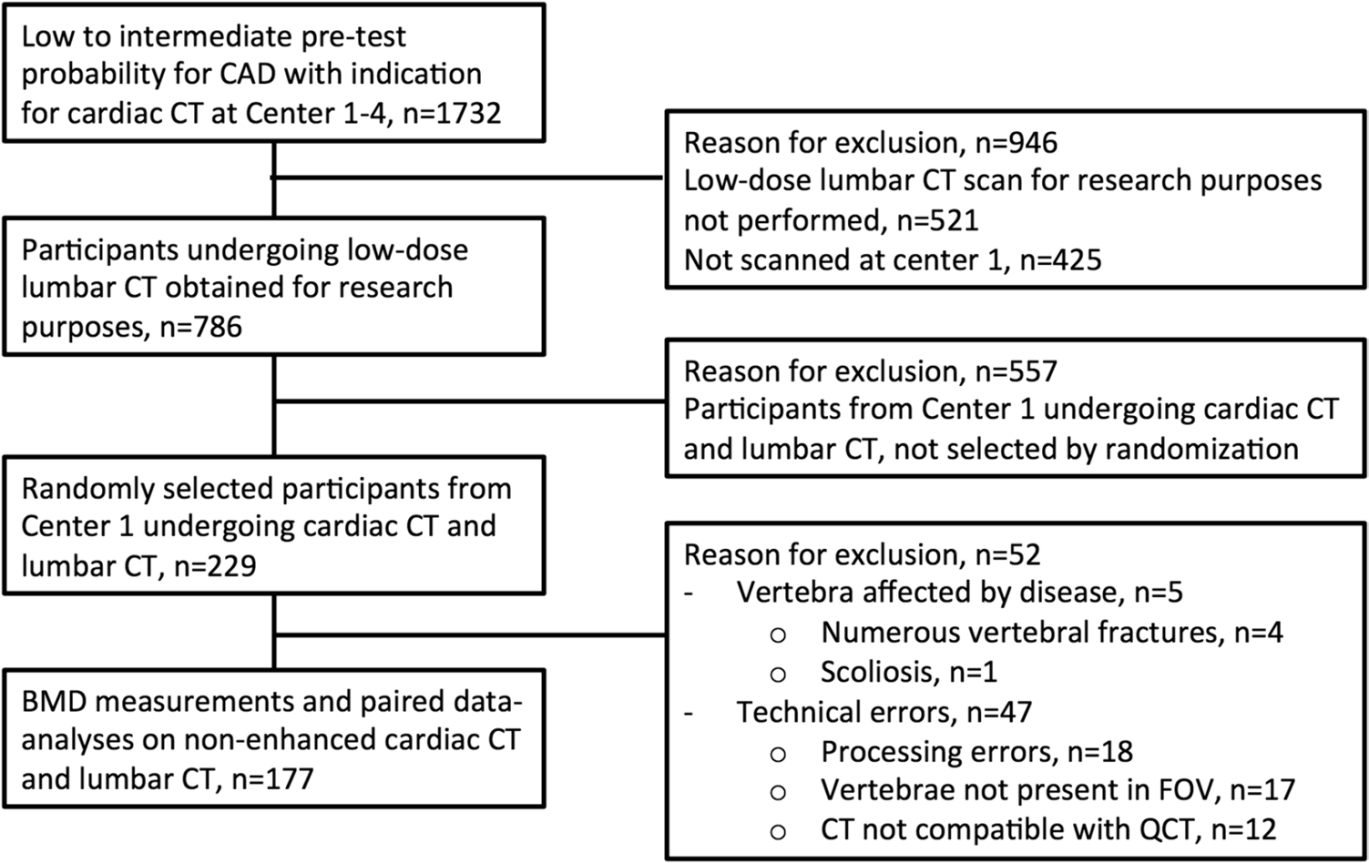
Participant inclusion flowchart. This sub-study is based on a large clinical cohort (*n* = 1732), included at four different centers. Detailed inclusion and exclusion criteria have previously been published [21]. In the current study, a total of 177 participants were included from the main study cohort for further BMD analyses. ‘Vertebrae not present in FOV’ refers to excluded CT scans in which the vertebrae were not fully included in the axial plane due to FOV < 500 mm. Abbr.: BMD, bone mineral density; CAD, coronary artery disease; CT, computed tomography; FOV, field of view; QCT, quantitative computed tomography

This cross-sectional, paired, single-center sub-study is based on a large clinical prospective cohort trial [20]. The study protocol has previously been reported in detail [21]. In short, patients with stable chest pain underwent coronary computed tomography (CT) angiography on a clinical indication, including a non-enhanced cardiac scan that opportunistically enabled BMD measurements of the thoracic vertebrae using QCT. As part of the study protocol, a non-enhanced lumbar CT scan including the vertebrae (L1–L3) was also obtained. Participants for this sub-study were chosen from the main study by a random allocation. Patients with conditions known to affect the QCT measuring method were excluded (Fig. 1). The reasons for excluding CT images due to technical errors included at random processing errors, such as failure to retrieve CT images from servers and errors related to converting file formats for QCT analyses. Furthermore, patients were also excluded if whole vertebrae were not included in the scan field of view. As the main focus of the original large clinical prospective cohort trial was evaluation of the heart, some cardiac CT scans did not include entire vertebrae and were therefore excluded from BMD analyses.

### CT Scan Protocol

All CT scans were conducted using a 320 multi-slice CT scanner (Aquillion One, Canon Medical Systems, Japan). The scan protocol has previously been published in detail [21]. In short, a non-contrast cardiac calcium score volume CT scan was performed initially. Subsequently, a low-dose helical abdominal CT scan without contrast was conducted. Both scans were performed with a tube voltage of 120 kilovoltage, with tube current and table height adjusted to patient size. Both scans had a gantry rotation time of 0.35 s but differed in total beam collimations of 160 × 0.5 mm and 80 × 0.5 mm, respectively (Supplementary Table S1). All images were reconstructed to a slice thickness of 2 mm using Philips Intellispace Portal 8.0. Additionally, the QCT Mindways Solid 3 phantom (Mindways Software Inc., TX) was scanned at regular intervals to ensure asynchronous calibration, scanner stability, and quality assurance. The scanner stability was tested at every asynchronous phantom scan calibration, and no scanner instability was detected in the study period. The study was approved by the local ethics committee and registered at clinical trials.gov identifier: NCT02264717.

### Vertebral Fracture Assessment

Vertebral fractures (VF) were assessed using 2-mm slice images from a sagittal and frontal view using a semi-quantitative technique first described by Genant et al. [22]. A trained reader (AC) reviewed and flagged vertebrae with ≥ 20% height reduction, which was subsequently confirmed by an experienced radiologist (CI). All VFs were excluded from BMD analyses. As described in ISCD official positions, other vertebrae can be used if the relevant lumbar vertebrae are not assessable for BMD measurements [12]. Therefore, in cases of VF or scan artifacts, the vertebra above or below was chosen as replacement to ensure the inclusion of three vertebrae at each measurement site.

### BMD Analyses

Volumetric BMD measurements of thoracic and lumbar CT scans were conducted using Mindways QCT Pro software 6.1 (Mindways Software Inc., TX). Thoracic BMD for each participant was calculated as the mean of three consecutive vertebrae (3TS) starting from the level of the left anterior descending artery expectedly corresponding to Th7 (48%), Th8 (37%), Th6 (11%), or Th9 (4%) [16]. Lumbar BMD for each participant was calculated as the mean of L1–L3 (3LS). L1 was identified as the first consecutive vertebra following the last thoracic rib-carrying vertebra (Supplementary Figure S1). Mindways software algorithm enabled each vertebra a 9-mm high elliptical volume of interest (VOI), which was automatically placed in the anterior ½–¾ part of the trabecular bone compartment. This was followed by visually adjusting VOI placement by one reader to encompass as much trabecular bone as possible while excluding cortical bone, osteophytes, and the posterior venous plexus (Supplementary Fig. S1). A mean BMD in mg/cm^3^ for all individual vertebrae and a mean of all three vertebrae were calculated for all participants.

### American College of Radiology QCT Classification

We used the American College of Radiology (ACR) QCT cut-off values for lumbar spine to categorize patients into ‘very low BMD’ (mean BMD value < 80 mg/cm^3^), ‘low BMD’ (mean BMD value between 80 and 120 mg/cm^3^), or ‘normal BMD’ (mean BMD value > 120 mg/cm^3^).

### Statistical Analyses

Normal distribution was tested using QQ-plots and histograms. Parametric variables are presented as mean ± standard deviation, and nonparametric variables as median with interquartile range. *XY* and Bland–Altman plots visualized correlations (Fig. 4) and any systematic bias (Supplementary Fig. S2). Paired *t* test was used to assess differences in BMD values between scan regions, and independent Students* t* test was used to assess dichotomous groups. One-way ANOVA, Kruskal–Wallis test, and *χ*^*2*^ test were used to test variables by groups. The coefficient of determination, *r*^*2*^, was used to express the linear regression between 3LS and 3TS by least square fit. BMD cut-off values were calculated by inserting the lumbar spine BMD cut-off values of 80 and 120 mg/cm^3^ into the linear regression equation. Statistical analyses were performed using the software package STATA/MP 17.0 (StataCorp LP, College Station, TX, USA).

A two-sided *p* value < 0.05 was considered statistically significant.

## Results

This study included a total of 177 participants, 90 (51%) were women, and the mean age was 61 years [interquartile range: 52–65]; age range 31–74 years, all with European ancestry. Baseline demographics stratified by BMD groups based on lumbar spine measurements are presented in Table 1.

**Table 1:** Baseline demographics by diagnostic group according to lumbar BMD

Characteristics	All (*n* = 177)	Lumbar BMD			*p* value
		Very low (*n* = 26)	Low (*n* = 62)	Normal (*n* = 89)	
Sex, women	90/177 (51%)	17/26 (65%)	28/62 (45%)	45/89 (51%)	0.97
Age, years	61 [52–65]	67 [63–70]	64 [59-6]	54 [49–61]	< 0.001
Height, cm	173 ± 10	173 ± 11	173 ± 10	174 ± 9	0.88
Weight, kg	82 ± 16	83 ± 18	79 ± 16	84 ± 16	0.19
Body Mass Index, kg/m^2^	27 ± 4	28 ± 4	26 ± 4	28 ± 4	0.09
Risk factors					
Smoking status(self-reported data at the baseline visit)					0.09
Never	80/177 (45%)	10/26 (38%)	31/62 (50%)	39/89 (44%)	
Former	32/177 (18%)	1/26 (4%)	12/62 (19%)	19/89 (21%)	
Active	65/177 (37%)	15/26 (60%)	19/62 (31%)	31/89 (35%)	
Cigarette pack years* (self-reported data at the baseline visit)	18 [7–30]	24 [19–39]	22 [7–40]	14 [6–22]	0.04
Diabetes mellitus** (self-reported data at the baseline visit)	10/177 (1%)	2/26 (8%)	4/62 (6%)	4/62 (4%)	0.15
Bone data					
DXA performed previously (self-reported data at the baseline visit)	29/177 (16%)	7/26 (27%)	15/62 (24%)	7/89 (8%)	< 0.001
Osteoporosis diagnosed previously (self-reported data at the baseline visit)	15/177 (8%)	3/26 (12%)	8/62 (13%)	4/89 (4%)	< 0.001
Family history of osteoporosis(self-reported data at the baseline visit)	35/177 (20%)	8/26 (31%)	12/62 (19%)	15/89 (17%)	0.37
Vitamin D and/or calcium(self-reported data at the baseline visit)	49/177 (28%)	9/26 (35%)	17/62 (27%)	23/89 (26%)	0.68

Categorical data are presented as numbers with % in parenthesis, normally distributed continuous variables as mean ± standard deviation, and skewed variables as median with interquartile range in bracket. Participants were grouped using the lumbar spine QCT-based categories defined by American College of Radiology as very low BMD (< 80 mg/cm^3^), low (80–120 mg/cm^3^), and normal (> 120 mg/cm^3^) BMD. One-way analysis of variance, Kruskal–Wallis test, and χ^2^ test were used to test variables by groups. Family history of osteoporosis included first-degree relatives with either known osteoporosis or a previous hip fracture

*Only participants who were former or active smokers. A cigarette pack year was defined as smoking one pack (containing 20 cigarettes) per day for 1 year

**Diabetes mellitus including type I and II

*BMD* bone mineral density;* QCT* quantitative computed tomography;* DXA* dual-energy X-ray absorptiometry

### Vertebral Fractures

As shown in Figs. 1, 4/229 (2%) cases of no lumbar vertebra were eligible for analysis due to VF and were excluded. Subsequently, 22/177 (12%) patients had at least one VF (≥ 20% height reduction) in thoracic or lumbar vertebrae of interest. At the thoracic site, 9/177 (5%) participants had ≥ 1 VF, and at the lumbar site, 21/177 (12%) participants had ≥ 1 VF. Mean BMD in participants with a VF was lower, however, not significant in both 3LS (120 ± 52 vs 122 ± 36, *p* = 0.83) and 3TS (127 ± 41 vs 139 ± 37 *p* = 0.17), compared to participants without a VF.

### Differences and Classification using Lumbar and Thoracic Vertebrae

**Fig. 2:**
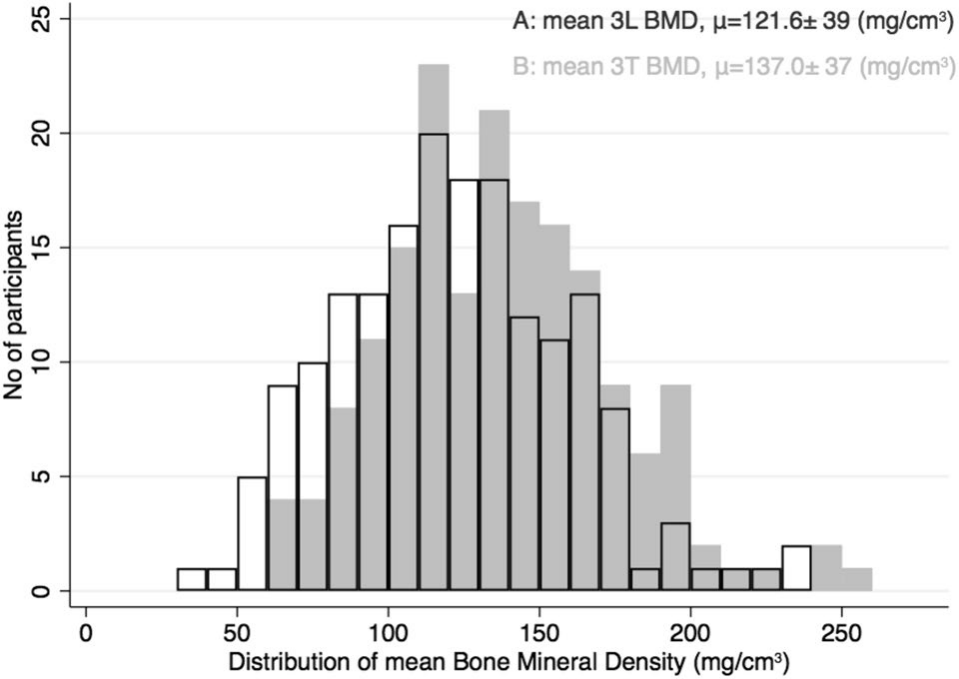
Illustration of lumbar and thoracic mean BMD for the total study cohort. All participants (*n* = 177) displayed according to their **a** mean lumbar BMD (black) and **b** mean thoracic BMD (gray). A: Lumbar BMD in black frames with a distribution mean of 121.6 ± 39 mg/cm^3^. Every value is a mean of BMD measured in L1–L3 (3LS), corresponding to three consecutive vertebrae from the last thoracic rib-carrying vertebra. **b** Thoracic BMD in gray bins with a distribution mean of 137.0 ± 37 mg/cm^3^*.* Lumbar and thoracic BMD distributions are significantly different by Student’s *t* test, *p* < *0.001*. Every value is a mean of 3TS, with the first vertebra corresponding to the level of the left anterior descending artery. Abbr.: BMD, bone mineral density; 3LS, lumbar spine BMD; 3TS, thoracic spine BMD

**Fig. 3:**
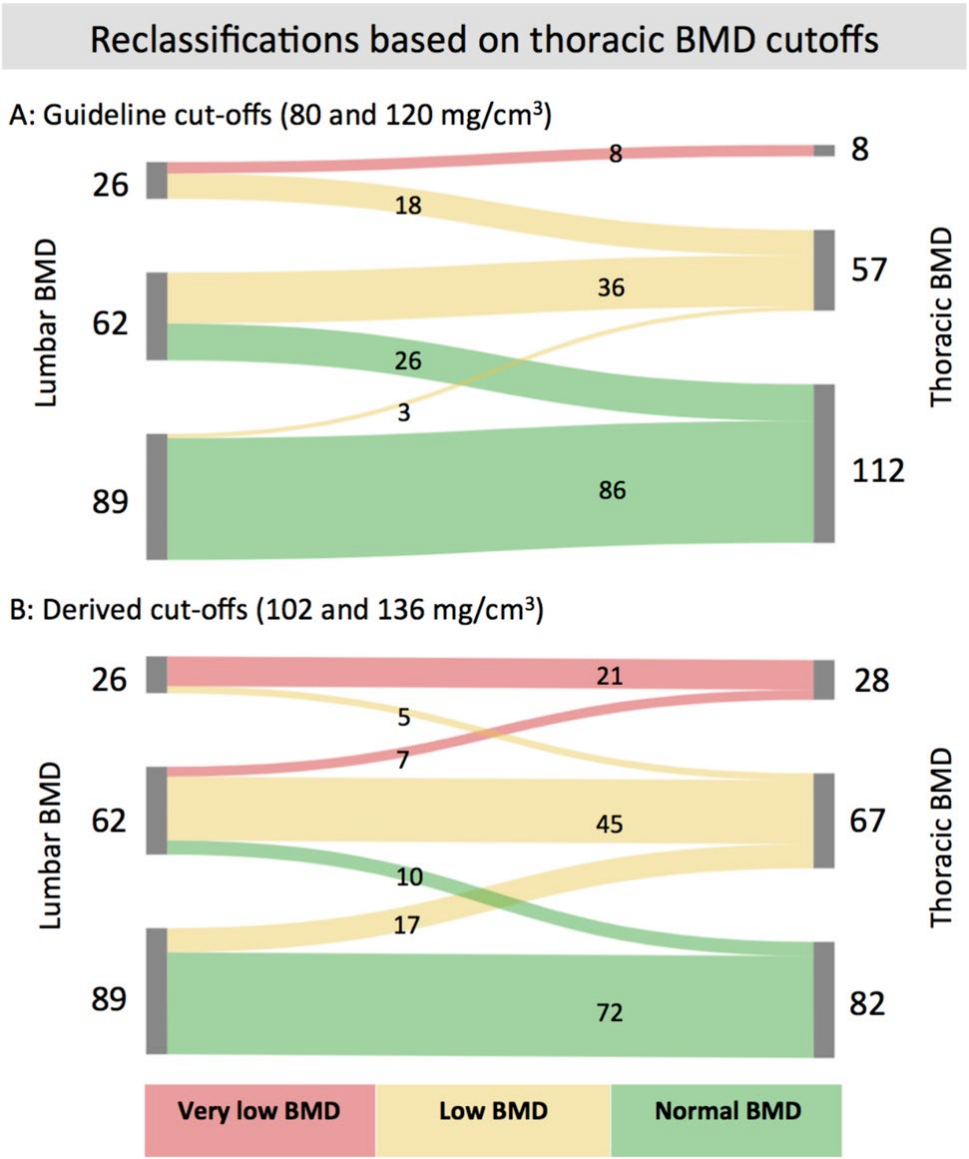
Illustration of participants being **a** reclassified to a different BMD category according to measurement site; **b** reclassifications of participants according to our proposed thoracic cut-off values. All participants were first classified according to BMD categories based on their mean lumbar spine BMD (3LS). (A) In total, 47/177 (27%) were reclassified based on their mean thoracic spine BMD (3TS) when applying current recommended cut-off values. Most participants (*n* = 44) changed into a less severe category, from very low to low (*n* = 18) or from low to normal (*n* = 26) BMD and a few (*n* = 3) changed categories from normal to low BMD. **b** Participants classified according to our derived thoracic cut-off values, resulting in 28/177 (16%) having very low BMD, 67/177 (38%) with low BMD, and 82/177 (46%) with normal BMD. This approximates the initial fractions based on the lumbar spine cut-off values. ACR cut-off values for lumbar spine BMD were very low (< 80 mg/cm^3^), low (80–120 mg/cm^3^), and normal BMD (> 120 mg/cm^3^) [14]. Our proposed cut-offs for thoracic spine BMD were very low (< 102 mg/cm^3^), low (102–136 mg/cm^3^), and normal BMD (> 136 mg/cm^3^). Abbr.: ACR, American College of Radiology; BMD, bone mineral density

Mean 3LS BMD was 121.6 mg/cm^3^ (95%CI 115.9–127.3) and mean 3TS BMD was 137.0 mg/cm^3^ (95%CI 131.5–142.5), *p* < 0.001 (Table 2) equivalent to a mean difference of 15.5 ± 18.7 mg/cm^3^. BMD distributions of 3LS and 3TS are illustrated in Fig. 2. Using the proposed ACR cut-off values for lumbar spine BMD values, 26/177 (14%) participants had very low BMD, 62 (35%) participants had low BMD, and 89 (45%) participants had normal BMD. Using the same cut-off values for thoracic spine BMD values, 47/177 (27%) participants were reclassified, of whom 44 (25%) changed into a less severe group. Of these, 18 (10%) participants with very low BMD were reclassified as having low BMD and 26 (15%) participants with low BMD was reclassified as having normal BMD (Fig. 3), whereas 8 out of 26 (31%) participants would still be identified as having very low BMD. A few (3/177) changed category to a more severe BMD group, i.e., from normal to low BMD.

**Table 2:** Age and sex stratification of bone mineral density assessed at the thoracic and lumbar spine

Characteristics					Women			Men		
	All (*n* = 177)	Age ≤ 60 (*n* = 88)	Age > 60 (*n* = 89)	*p* value	Age ≤ 60 (*n* = 45)	Age > 60 (*n* = 45)	*p* value	Age ≤ 60 (*n* = 43)	Age > 60 (*n* = 44)	*p* value
Thoracic BMD (mg/cm^3^)										
Vertebra 1	137 ± 41	149 ± 44	125 ± 35	< 0.001	145 ± 46	112 ± 30	< 0.001	142 ± 40	138 ± 34	0.61
Vertebra 2	135 ± 41	149 ± 37	120 ± 39	< 0.001	156 ± 41	109 ± 34	< 0.001	142 ± 32	132 ± 41	0.19
Vertebra 3	134 ± 44	148 ± 47	120 ± 36	< 0.001	157 ± 53	116 ± 28	< 0.001	138 ± 39	125 ± 41	0.14
Thoracic_(mean)_	137 ± 37	151 ± 37	123 ± 32	< 0.001	159 ± 40	113 ± 29	< 0.001	143 ± 32	134 ± 33	0.20
Lumbar BMD (mg/cm^3^)										
L1	125 ± 40	141 ± 38	109 ± 36	< 0.001	149 ± 44	101 ± 30	< 0.001	133 ± 28	117 ± 40	0.04
L2	119 ± 44	139 ± 41	100 ± 38	< 0.001	144 ± 51	93 ± 29	< 0.001	134 ± 26	106 ± 45	< 0.001
L3	106 ± 47	123 ± 46	89 ± 41	< 0.001	122 ± 56	84 ± 29	< 0.001	124 ± 34	94 ± 52	< 0.01
Lumbar_(mean)_	121.6 ± 38.5	140 ± 35	104 ± 34	< 0.001	148 ± 39	93 ± 28	< 0.001	132 ± 27	115 ± 35	0.02
Absolute difference between thoracic and lumbar (ΔBMD = Thoracic − Lumbar) and relative difference (ΔBMD = Thoracic/Lumbar)										
Absolute ΔBMD (mg/cm^3^)	16 ± 19, *p* < *0.001*	11 ± 20	20 ± 16	< 0.01	11 ± 22	20 ± 13	0.02	12 ± 19	19 ± 19	0.07
Relative ΔBMD	1.16 ± 0.20, *p* < *0.001*	1.09 ± 0.15	1.23 ± 0.21	< 0.001	1.08 ± 0.15	1.25 ± 0.21	< 0.001	1.09 ± 0.15	1.21 ± 0.21	< 0.01

Data are presented as normally distributed continuous variables with mean ± standard deviation. Participants were grouped by age ≤ 60 years (range: 31–60) and age > 60 years (range: 61–74) which ensured an approximately equal number of participants in the two groups. Individual vertebrae in the thoracic spine, (3TS), corresponds to the three consecutively included vertebrae starting at the level of the left anterior descending artery. Individual vertebrae in the lumbar spine, L1–L2–L3 (3LS), corresponds to the first, second, and third lumbar vertebra from the last thoracic rib-carrying vertebra. Student’s *t* test was used to assess differences in BMD values between scan regions and to test dichotomous groups

*BMD* bone mineral density

### Stratification by Age and Sex

Baseline data stratified by age and sex, as well as BMD values for individual vertebrae are presented in Table 2 and Supplementary Table S2. All BMD measurements at 3LS and 3TS were found inversely correlated to age (Tables 2 and S3). The mean difference between 3LS and 3TS BMD values in the age group ≤ 60 was 11.2 mg/cm^3^ (95%CI 6.9–15.5), whereas mean difference in the age group > 60 was 19.7 mg/cm^3^ (95%CI 16.3–24.1), *p* < *0.01*. Age stratification showed that women ≤ 60 years had higher BMD values compared to men of the same age across all vertebral levels and when comparing sites (mean difference: 159 ± 40 vs. 143 ± 32 thoracic, *p* < *0.01*; 148 ± 39 vs. 132 ± 27 lumbar, *p* < *0.01*), whereas women > 60 years had lower BMD values than men, also across all levels (mean difference: 113 ± 29 vs. 134 ± 33 thoracic, *p* < *0.01*, 93 ± 28 vs. 115 ± 35 lumbar, *p* < *0.01*). BMD measurements stratified by sex alone did not show significant differences in either individual levels or sites (136 ± 42 vs. 139 ± 32, thoracic p = 0.61; 120 ± 44 vs. 123 ± 32, lumbar *p* = 0.61), as presented in Supplementary Table S4.

### Thoracic Spine BMD Cut-off Values

**Fig. 4:**
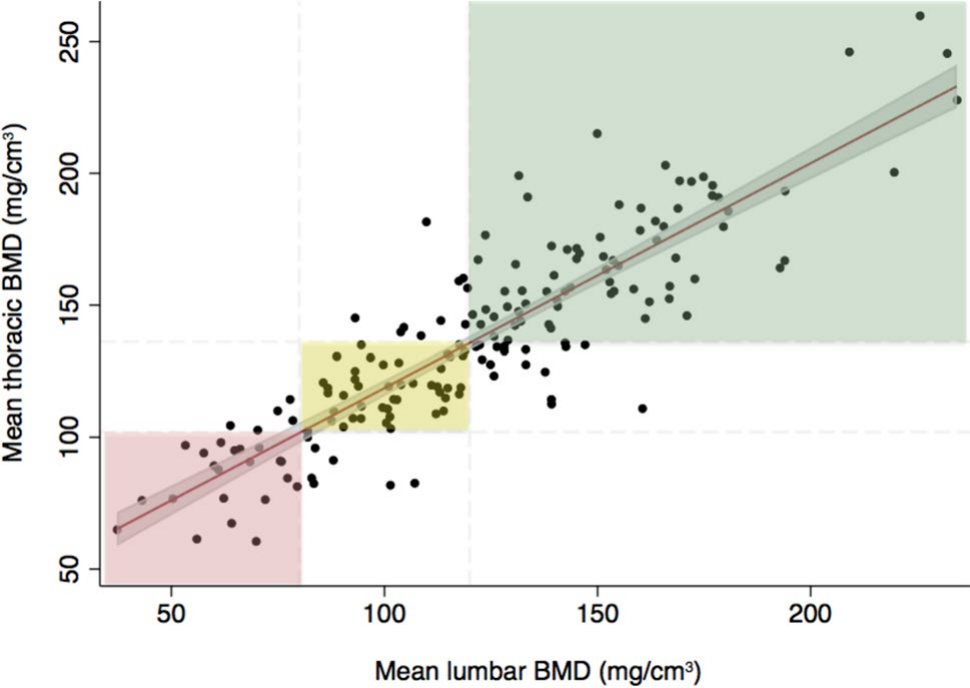
The association between lumbar BMD and thoracic BMD for the total study cohort (*n* = 177). Every datapoint represents a participant. Scatter plot with linear regression: $y=0.85{x}+33.5, {{r}}^{2}=0.77,$ and 95% CI (gray shadow). Participants in the red zone are categorized as very low BMD according to lumbar, thoracic, or both sites; participants in the yellow zone as having low BMD; and participants in the green zone as having normal BMD. Zones are based on the following cut-off values: lumbar spine BMD values as recommended by the American College of Radiology: very low BMD (< 80 mg/cm^3^), low (80–120 mg/cm^3^), and normal (> 120 mg/cm^3^) BMD [14]. The thoracic values are derived by calculating the thresholds for very low cut-off: $tBMD=0.85*80+33.5\approx 102$ and low cut-off: $tBMD=0.85*120+33.5\approx 136$ according to our linear regression. Abbr.: BMD, bone mineral density; CI, confidence interval; *r*^2^, coefficient of determination; QCT, quantitative computed tomography

Using linear regression analyses, 3LS and 3TS BMD values had a coefficient of determination of *r*^*2*^ = *0.77* (Fig. 4). Proposals for adjusted cut-off values for thoracic BMD values derived from lumbar categories were defined as very low BMD corresponding to a mean BMD < 102 mg/cm^3^, low BMD as a mean BMD between 102 and 136 mg/cm^3^, and normal BMD as a mean BMD > 136 mg/cm^3^. These assumptions are based on calculating the linear regression equations: $\text{tBMD}=0.85*80+33.5\approx 102$ and $\text{tBMD}=0.85*120+33.5\approx 136$. This resulted in a measurement agreement between lumbar BMD categorized by ACR classifications, and thoracic BMD categorized by our derived cut-off values of 89% and a kappa-value of 0.71 using weighted statistic (Fig. 3).

## Discussion

This paper aimed to quantify and describe the discrepancies in BMD values between the lumbar spine, which is the current standard QCT BMD measurement site, and the thoracic spine as included in routine cardiac CT scans. These measurements enabled development of thoracic spine BMD cut-off values derived from lumbar spine cut-offs. Our findings revealed statistically significant differences in mean BMD values between the regions, resulting in a substantial reclassification of participants across diagnostic categories depending on the reference site. However, the thoracic and lumbar BMD values demonstrated a linear relationship, which formed the basis of our suggested cut-off values for low BMD detection based on the thoracic spine BMD values.

### Previous Studies on Thoracic and Lumbar BMD

Studies proposing methods for diagnosing osteoporosis using thoracic spine BMD are sparse. For lumbar spine, Mao et al. reported ≈8% higher mean BMD value, but for thoracic spine, the reported mean BMD value was ≈26% higher [23]. Though Mao et al. used a similar method in QCT analyses, including the software and selection of specific thoracic and lumbar vertebrae, the scan indications differed; Mao et al. included 121 asymptomatic patients, whereas our study cohort of 177 participants underwent cardiac CT based on symptoms suggestive of obstructive coronary artery disease (CAD). Our overall lower mean BMD values could be explained by the link between CAD and decreased BMD described as the ‘calcification paradox’ [24]. Importantly, participants in this study were on average 5 years older (mean age of 61, range 30–75 years) and all with European ancestry, compared to Mao et al. which included a population of 50% African, 30% Hispanic, 10% Asian, 5% Caucasian with a mean age of 56 years, range 20–81 years. Variations in mean BMD values between ethnicities exist, differing 13–40% depending on body site and sex [17, 25]. Budoff et al. [15] assessed thoracic BMD in 9585 asymptomatic participants, for which he developed QCT-based thoracic* T*-scores based on a subgroup of 95 healthy participants with a mean age of 30 years. They posed as reference to calculate standard deviations for the rest of the study population, mimicking the current method of DXA based* T*-scores on the lumbar spine. However, study cohort demographic and enrollment criteria differed. The study by Budoff et al. included asymptomatic participants with a mixed ethnicity, whereas this study enrolled individuals with European ancestry only and with symptoms suggestive of CAD. These differences likely contribute to the differences in mean BMD observed between the two studies.

### Age and Sex Stratification

An inverse association between BMD and age was found in our data and is previously well described [16, 23, 25]. Interestingly, we found lumbar BMD values to decrease by age more than thoracic BMD values. Budoff et al. also documented this [16], whereas others found lumbar and thoracic values to decrease by age at similar rates [23, 25]. Age-related bone loss is primarily seen in the trabecular bone compartment and can be attributed to factors, such as estrogen deficiency in women [26] and lack of mechanical burdens, e.g., immobilization [27].

The average trabecular BMD loss measured by QCT is estimated to be 8–10 mg/cm^3^ per year in postmenopausal women [28], whereas men have a bone loss reported to be approximately 20% less in the trabecular compartment [29]. Not surprisingly, studies report significant differences in BMD when stratified by sex [15, 30]. Areal BMD is known to be dependent on bone size, which varies between men and women, likely contributing to the significant differences in BMD as measured by DXA [31]. However, the same limitation does not apply to volumetric BMD as measured by QCT. This likely contributed to our findings, with no overall differences in mean BMD between men and women. Significant differences were observed only in our cohort when stratifying BMD by both age and sex; this observation can be attributed to the study cohort, including pre-menopausal women without increased bone loss following menopause. Conversely, women above the age of 60 had significantly lower BMD levels than men in the same age group, possibly leveling out the existing differences between men and women in our study. Therefore, this study only reported cut-off values for both men and women combined, in line with the proposed cut-off values by ACR [14]. A previous study has also reported no overall significant difference in QCT-measured BMD values between men and women [32]. However, more research is needed, especially considering the BMD evaluation in premenopausal women and men younger than 50 years of age.

### Clinical Implications

The thoracic CT scan field of view (FOV) ensured inclusion of the relevant measurement area, and thoracic BMD was obtained as a mean of three consecutive descending vertebras, starting from the level of the LAD. This method ensures consistency in the selected vertebra between individuals despite anatomic varieties [15, 18, 25]. The use of three vertebrae for QCT measurements was chosen to ensure comparability with previous QCT-based studies [12, 15, 23] and to align with the ACR official paper [14]. All cardiac CT scans consistently included three vertebrae, which aligned with our methodology aim to include three vertebrae at both the thoracic and lumbar spine. We chose not to calculate threshold values for each individual vertebra, as the mean of multiple vertebrae likely provided the most reliable estimate due to lower risk of random measurement errors. The predictive value of very low thoracic BMD values for incident osteoporotic-related and any type of fractures have previously been documented in a longitudinal study [19]. Therkildsen et al. also included patients with European ancestry only, which were referred to cardiac CT based on symptoms. This study had a median follow-up period of 3.1 years and in a cohort of 1487 participants, they found an optimal cut-off of 103 mg/cm^3^ to predict any type of fracture. This is comparable to the thoracic cut-off value developed in this study (102 mg/cm^3^) based on a different study cohort referred to routine cardiac CT, to categorize very low thoracic BMD. In the adjusted classifications, some participants changed category depending on the body site being measured, with 12/177 (7%) being in between the categories of very low and low BMD. Potentially diagnosing a greater number of patients is a trade-off involving an increased disease-burden on individuals, heightened costs, and the potential for unnecessary treatment.

### QCT Software Programs, CT Scanners, Calibration, and Reproducibility

We conducted BMD measurements using Mindways QCT Pro, but previous QCT-based studies use several different software programs for BMD estimation. Knowledge regarding potential measurement bias by analyzing BMD with different software programs is currently limited, and more studies are encouraged to address the matter of potential variability in BMD estimates between different software solutions. The accuracy of the QCT methodology highly relies on the calibration protocol for the conversion of Hounsfield Units to BMD units and for monitoring scanner stability. In our study, we chose asynchronous calibration, which is an established calibration method for QCT [33, 34]. The reproducibility of our thoracic QCT measurements using asynchronous calibration, previously published in detail [35] with an inter-reader CV of 2.1% and an intra-reader CV of 1.5%, was comparable with previous studies [20]. Another aspect is the potential variations in performance across various vendors and models of CT scanners. Different CT scanners use various techniques to reduce radiation and to obtain the best image quality [36]. As an example, newer CT systems enable use of scan with lower kilovoltage. These techniques are not developed to optimized measurements of BMD and it would be expected that a model-specific calibration is necessary [37].

### Future Perspectives

External validation of our proposed thoracic cut-off values is of essential matter, and several considerations remains for future implication. We developed and tested the thoracic cut-off values in the same study population. We also related our proposed cut-off value to an independent but rather similar cohort of patients with European ancestry referred to cardiac CT based on symptoms [19]. Ideally, the cut-off development and testing should be conducted in an independent study population and in different study populations to strengthen the external validity. Another potential step would be a prospective cohort study assessing the assigned lumbar and thoracic BMD categorizations individually against clinical endpoints of any type of fractures and osteoporotic-related fractures. This would allow for a direct comparison between our proposed thoracic BMD cut-off values and the established lumbar spine QCT guidelines. Recently, the impact of contrast enhancement on BMD measurements in thoracic CT was assessed and revealed an overestimation of BMD because of contrast. However, this overestimation was also described by a linear relationship. A study investigating the development of cut-off values on contrast-enhanced thoracic CT scans could further increase the number of investigations eligible for BMD assessment [38].

### Strengths and Limitations

All participants were included at a single center, scanned with an identical scan protocol, scanner type, and scanner settings. All BMD analyses were conducted by the same reader, although a high inter-reader variability have been demonstrated previously [38]. These measures ensured a homogeneous study population and uniform analyses. Since this is the first study proposing QCT cut-off values for thoracic BMD categories, we prioritized this setting to strengthen the internal validation.

Limitations include the number of excluded participants due to technical errors (*n* = 47, 27%) which was high; however, we consider the exclusion to be at random and independent of BMD status. Therefore, we do not consider the exclusion to have major impact on the findings. Our study included merely a Caucasian population, all scanned with a clinical indication of chest pain symptoms, which does not allow for generalizability to other ethnic groups. Lastly, we did not compare our results with the current gold standard DXA for diagnosing osteoporosis.

## Conclusion

In conclusion, our findings revealed a linear relationship between the significantly different thoracic and lumbar BMD values, which enabled the suggested cut-offs for normal, low and very low BMD detection in the thoracic spine. These proposed cut-off values need external validation, but they could potentially strengthen the use of opportunistic screening for osteoporosis as thoracic CT is extremely frequently used.

## Supplementary Information

Below is the link to the electronic supplementary material.

Supplementary file1 (PDF 1451 KB)

## Data Availability

The data supporting the results of this study is not publicly available due to GDPR regulations.

## Abbreviations

3LS: Lumbar spine BMD
3TS: Thoracic spine BMD
ACR: American College of Radiology
ANOVA: Analysis of variance
BMD: Bone mineral density
CAD: Coronary artery disease
CI: Confidence interval
CT: Computed tomography
CV: Coefficient of variation
DXA: Dual-energy X-ray absorptiometry
FOV: Field of view
HU: Hounsfield units
ICC: Intraclass correlation coefficient
IQR: Interquartile range
QCT: Quantitative computed tomography
SD: Standard deviation
VOI: Volume of interest

## Declarations

### Human and Animal Participants

This is an original, clinical research paper involving human subjects who provided informed written consent.

## References

[1] Johnell O, Kanis JA (2006) An estimate of the worldwide prevalence and disability associated with osteoporotic fractures. Osteoporosis Int 17:1726–1733 10.1007/s00198-006-0172-4 16983459

[2] Schuit SC, van der Klift M, Weel AE, de Laet CE, Burger H, Seeman E, Hofman A, Uitterlinden AG, van Leeuwen JP, Pols HA (2004) Fracture incidence and association with bone mineral density in elderly men and women: the Rotterdam Study. Bone 34:195–202 14751578 10.1016/j.bone.2003.10.001

[3] LeBoff MS, Greenspan SL, Insogna KL, Lewiecki EM, Saag KG, Singer AJ, Siris ES (2022) The clinician’s guide to prevention and treatment of osteoporosis. Osteoporosis Int 33:2049–2102 10.1007/s00198-021-05900-y PMC9546973 35478046

[4] Cosman F, de Beur SJ, LeBoff MS, Lewiecki EM, Tanner B, Randall S, Lindsay R (2014) Clinician’s guide to prevention and treatment of osteoporosis. Osteoporosis Int 25:2359–2381 10.1007/s00198-014-2794-2 PMC4176573 25182228

[5] Ott SM, O'Hanlan M, Lipkin EW, Newell-Morris L (1997) Evaluation of vertebral volumetric vs. areal bone mineral density during growth. Bone 20:553–556. 10.1016/s8756-3282(97)00057-4 9177870

[6] Link TM (2012) Osteoporosis imaging: state of the art and advanced imaging. Radiology 263:3–17 22438439 10.1148/radiol.12110462 PMC3309802

[7] Engelke K (2017) Quantitative computed tomography-current status and new developments. J Clin Densitom 20:309–321. 10.1016/j.jocd.2017.1006.1017. 10.1016/j.jocd.2017.06.017 28712984

[8] Liu G, Peacock M, Eilam O, Dorulla G, Braunstein E, Johnston CC (1997) Effect of osteoarthritis in the lumbar spine and hip on bone mineral density and diagnosis of osteoporosis in elderly men and women. Osteoporosis Int 7:564–569 10.1007/BF02652563 9604053

[9] Lin W, He C, Xie F, Chen T, Zheng G, Yin H, Chen H, Wang Z (2023) Quantitative CT screening improved lumbar BMD evaluation in older patients compared to dual-energy X-ray absorptiometry. BMC Geriatr 23:231 37069511 10.1186/s12877-023-03963-6 PMC10108496

[10] Brown JK, Timm W, Bodeen G, Chason A, Perry M, Vernacchia F, DeJournett R (2017) Asynchronously calibrated quantitative bone densitometry. J Clin Densitom 20:216–225 26781430 10.1016/j.jocd.2015.11.001

[11] Lenchik L, Weaver AA, Ward RJ, Boone JM, Boutin RD (2018) Opportunistic screening for osteoporosis using computed tomography: state of the art and argument for paradigm shift. Curr Rheumatol Rep 20:74 30317448 10.1007/s11926-018-0784-7 PMC7092507

[12] Engelke K, Adams JE, Armbrecht G, Augat P, Bogado CE, Bouxsein ML, Felsenberg D, Ito M, Prevrhal S, Hans DB, Lewiecki EM (2008), Clinical use of quantitative computed tomography and peripheral quantitative computed tomography in the management of osteoporosis in adults: the 2007 ISCD Official Positions. J Clin Densitom 11(2008):123–162. 10.1016/j.jocd.2007.1012.1010 10.1016/j.jocd.2007.1012.1010 18442757

[13] Adams JE (2013) Advances in bone imaging for osteoporosis, nature reviews. Endocrinology 9:28–42 23232496 10.1038/nrendo.2012.217

[14] A.C.o. Radiology, ACR–SPR–SSR Practice parameter for the performance of musculoskeletal quantitative computed tomographY (QCT), Revised 2018 (Resolution 9), (2018) 14.

[15] Budoff MJ, Hamirani YS, Gao YL, Ismaeel H, Flores FR, Child J, Carson S, Nee JN, Mao S (2010) Measurement of thoracic bone mineral density with quantitative CT. Radiology 257:434–440 20807844 10.1148/radiol.10100132

[16] Budoff MJ, Khairallah W, Li D, Gao YL, Ismaeel H, Flores F, Child J, Carson S, Mao SS (2012) Trabecular bone mineral density measurement using thoracic and lumbar quantitative computed tomography. Acad Radiol 19:179–183 22112461 10.1016/j.acra.2011.10.006

[17] Wong M, Papa A, Lang T, Hodis HN, Labree L, Detrano R (2005) Validation of thoracic quantitative computed tomography as a method to measure bone mineral density. Calcif Tissue Int 76:7–10 15455185 10.1007/s00223-004-0020-5

[18] Therkildsen J, Winther S, Nissen L, Jørgensen H, Thygesen J, Ivarsen P, Frost L, Langdahl B, Hauge E, Bøttcher M (2018) Feasibility of opportunistic screening for low thoracic bone mineral density in patients referred for routine cardiac CT. J Clin Densitom, p 23. 10.1016/j.jocd.2018.12.002 30665819

[19] Therkildsen J, Nissen L, Jørgensen HS, Thygesen J, Ivarsen P, Frost L, Isaksen C, Langdahl BL, Hauge EM, Boettcher M, Winther S (2020) Thoracic bone mineral density derived from cardiac CT is associated with greater fracture rate. Radiology 296:499–508 32662758 10.1148/radiol.2020192706

[20] Rasmussen LD, Fordyce CB, Nissen L, Hill CL Jr, Alhanti B, Hoffmann U, Udelson J, Bøttcher M, Douglas PS, Winther S (2022) The PROMISE minimal risk score improves risk classification of symptomatic patients with suspected CAD, JACC. Cardiovascular Imag 15:1442–1454 10.1016/j.jcmg.2022.03.009 35926903

[21] Rasmussen LD, Winther S, Westra J, Isaksen C, Ejlersen JA, Brix L, Kirk J, Urbonaviciene G, Søndergaard HM, Hammid O, Schmidt SE, Knudsen LL, Madsen LH, Frost L, Petersen SE, Gormsen LC, Christiansen EH, Eftekhari A, Holm NR, Nyegaard M, Chiribiri A, Bøtker HE, Böttcher M (2019) Danish study of non-invasive testing in Coronary Artery Disease 2 (Dan-NICAD 2): study design for a controlled study of diagnostic accuracy. Am Heart J 215:114–128 31323454 10.1016/j.ahj.2019.03.016

[22] Genant HK, Wu CY, van Kuijk C, Nevitt MC (1993) Vertebral fracture assessment using a semiquantitative technique. J Bone Miner Res 8:1137–1148 8237484 10.1002/jbmr.5650080915

[23] Mao SS, Li D, Syed YS, Gao Y, Luo Y, Flores F, Child J, Cervantes M, Kalantar-Zadeh K, Budoff MJ (2017) Thoracic Quantitative Computed Tomography (QCT) can sensitively monitor bone mineral metabolism: comparison of thoracic QCT vs lumbar QCT and dual-energy X-ray absorptiometry in detection of age-relative change in bone mineral density. Acad Radiol 24:1582–1587 28844601 10.1016/j.acra.2017.06.013

[24] Szulc P (2016) Abdominal aortic calcification: a reappraisal of epidemiological and pathophysiological data. Bone 84:25–37 26688274 10.1016/j.bone.2015.12.004

[25] Li D, Mao SS, Khazai B, Hyder JA, Allison M, McClelland R, de Boer I, Carr JJ, Criqui MH, Gao Y, Budoff MJ (2013) Noncontrast cardiac computed tomography image-based vertebral bone mineral density: the multi-ethnic study of atherosclerosis (MESA). Acad Radiol 20:621–627 23570937 10.1016/j.acra.2013.01.007 PMC3622214

[26] Khosla S, Melton LJ 3rd, Riggs BL (2011) The unitary model for estrogen deficiency and the pathogenesis of osteoporosis: is a revision needed? J Bone Miner Res 26:441–451 20928874 10.1002/jbmr.262 PMC3179298

[27] Forwood MR (2001) Mechanical effects on the skeleton: are there clinical implications? Osteoporosis Int 12:77–83 10.1007/s001980170161 11305087

[28] Ettinger B, Genant HK, Cann CE (1987) Postmenopausal bone loss is prevented by treatment with low-dosage estrogen with calcium. Ann Intern Med 106:40–45 3789576 10.7326/0003-4819-106-1-40

[29] Yu W, Qin M, Xu L, van Kuijk C, Meng X, Xing X, Cao J, Genant HK (1999) Normal changes in spinal bone mineral density in a Chinese population: assessment by quantitative computed tomography and dual-energy X-ray absorptiometry. Osteoporosis Int 9:179–187 10.1007/s001980050133 10367047

[30] Carr JJ, Register TC, Hsu FC, Lohman K, Lenchik L, Bowden DW, Langefeld CD, Xu J, Rich SS, Wagenknecht LE, Freedman BI (2008) Calcified atherosclerotic plaque and bone mineral density in type 2 diabetes: the diabetes heart study. Bone 42:43–52 17964237 10.1016/j.bone.2007.08.023 PMC2239236

[31] Bruno AG, Broe KE, Zhang X, Samelson EJ, Meng CA, Manoharan R, D’Agostino J, Cupples LA, Kiel DP, Bouxsein ML (2014) Vertebral size, bone density, and strength in men and women matched for age and areal spine BMD. J Bone Mineral Res 29:562–569 10.1002/jbmr.2067 PMC4149904 23955966

[32] Buenger F, Eckardt N, Sakr Y, Senft C, Schwarz F (2021) Correlation of bone density values of quantitative computed tomography and hounsfield units measured in native computed tomography in 902 vertebral bodies. World Neurosurg 151:e599–e606 33933695 10.1016/j.wneu.2021.04.093

[33] Engelke K, Lang T, Khosla S, Qin L, Zysset P, Leslie WD, Shepherd JA, Shousboe JT (2015) Clinical Use of Quantitative Computed Tomography-Based Advanced Techniques in the Management of Osteoporosis in Adults: the 2015 ISCD Official Positions-Part III. J Clin Densitom 18:393–407 26277853 10.1016/j.jocd.2015.06.010

[34] Bauer JS, Henning TD, Müeller D, Lu Y, Majumdar S, Link TM (2007) Volumetric quantitative CT of the spine and hip derived from contrast-enhanced MDCT: conversion factors. AJR Am J Roentgenol 188:1294–1301 17449773 10.2214/AJR.06.1006

[35] Cheneymann A, Therkildsen J, Winther S, Nissen L, Thygesen J, Langdahl BL, Hauge EM, Bøttcher M (2024) Bone mineral density derived from cardiac CT scans: using contrast enhanced scans for opportunistic screening. J Clin Densitom 27:101441 38006641 10.1016/j.jocd.2023.101441

[36] Yu L, Liu X, Leng S, Kofler JM, Ramirez-Giraldo JC, Qu M, Christner J, Fletcher JG, McCollough CH (2009) Radiation dose reduction in computed tomography: techniques and future perspective. Imaging Med 1:65–84 22308169 10.2217/iim.09.5 PMC3271708

[37] Therkildsen J, Thygesen J, Winther S, Svensson M, Hauge EM, Böttcher M, Ivarsen P, Jørgensen HS (2018) Vertebral bone mineral density measured by quantitative computed tomography with and without a calibration phantom: a comparison between 2 different software solutions. J Clin Densitom 21:367–374 29680671 10.1016/j.jocd.2017.12.003

[38] Cheneymann A, Therkildsen J, Winther S, Nissen L, Thygesen J, Langdahl BL, Hauge EM, Bøttcher M (2023) Bone mineral density derived from cardiac CT scans: using contrast enhanced scans for opportunistic screening. J Clin Densitom, p 101441. 10.1016/j.jocd.2023.101441 38006641

