# Supplementary material for: Developing Cut-off Values for Low and Very Low Bone Mineral Density at the Thoracic Spine Using Quantitative Computed Tomography
Source: Calcif Tissue Int. 2024 Aug 16;115(4):421–31. doi: 10.1007/s00223-024-01268-3 (PMC11405482; doi:10.1007/s00223-024-01268-3)
Supplement: Supplementary file 1 — Supplementary file1 (PDF 1451 KB) [file 223_2024_1268_MOESM1_ESM.pdf]

Supplementary Material, Calcified Tissue International

Developing Cut-off Values for Low and Very Low Bone Mineral Density at the Thoracic Spine Using Quantitative Computed Tomography

Andia Cheneymann[a,b], Josephine Therkildsen[b,c], Laust Dupont Rasmussen[a,d], Jesper Thygesen[e], Christin Isaksen[f], Ellen-Margrethe Hauge[b,c], Simon Winther[a], Morten Böttcher[a,c]

[a]Department of Cardiology, University Clinic for Cardiovascular Research, Gødstrup Hospital, Hospitalsparken 15, Herning, Denmark

[b]Department of Rheumatology, Aarhus University Hospital, Palle Juul-Jensens Blvd. 99, Aarhus, Denmark

[c]Department of Clinical Medicine, Aarhus University, Palle Juul-Jensens Blvd. 11, Aarhus, Denmark

[d]Department of Cardiology, Aalborg University Hospital, Hobrovej 18-22, Aalborg, Denmark

[e]Department of Clinical Engineering, Aarhus University Hospital, Aarhus, Denmark

[f]Department of Radiology, Silkeborg Hospital, Falkevej 1D, Silkeborg, Denmark

Corresponding author

Morten Bøttcher Prof. MD PhD,

Department of Cardiology,

University Clinic for Cardiovascular Research Aarhus University / Gødstrup Hospital

Hospitalsparken 15, 7400 Herning, Denmark

E-mail: morboett@rm.dk

Phone: +45 7843 0000

Supplementary tables

Supplementary Table S1: CT scan protocols

	**Thoracic CT**	**Lumbar CT**	**Phantom scan**
Type	Volume CT	Helical CT	Volume CT
Purpose of scan	Cardiac calcium-score	Bone standard	QCT asynchronous calibration
Tube voltage, kilovoltage	120	120	120
Tube current	Dependent of patient size	Dependent of patient size	mA 200, Eff. mAs 100
Slice collimation	128 x 0.5 mm	80 x 0.5 mm	80 x 0.5 mm
Gantry rotation	0.35 seconds	0.35 seconds	0.5 seconds
Kernel	FC12	FC35	None
Scan field-of-view	500 mm	500 mm	320 mm
Table height	Patient size	Patient size	90 cm

Scanner: 320 multi-slice CT scanner (Aquillion One, Canon Medical Systems, Japan).

Supplementary Table S2: Baseline demographics stratified by age and sex

**Baseline data**		**Age stratification**			**Sex stratification**		
**Characteristics**	**All (n=177)**	**Age≤60 (n=88)**	**Age>60 (n=89)**	***p value***	**Women (n=90)**	**Men (n=87)**	***p value***
Sex, women	90/177 (51%)	45/88 (51%)	45/89 (51%)	0.94			
Age, years	61 [52–65]	52 [48–57]	65 [63–69]	<0.001	61 [52–65]	61 [53–65]	0.33
Height, cm	173±10	174.5±10	171.9±9	0.07	166±7	180±7	<0.001
Weight, kg	82±16	85±17	78±15	<0.01	75±14	90±15	<0.001
Body Mass Index, kg/m^2	27±4	28±4	26±4	0.01	27±5	28±4	0.37
**Risk factors**							
Smoking status				0.04			0.21
Never	80/177 (45%)	32/88 (36%)	33/89 (37%)		35/90 (39%)	45/87 (52%)	
Former	32/177 (18%)	22/88 (25%)	10/89 (11%)		17/90 (19%)	15/87 (17%)	
Active	65/177 (37%)	34/88 (39%)	46/89 (52%)		38/90 (42%)	27/87 (31%)	
Cigarette pack years*	18 [7–30]	13 [6–22]	22 [10–35]	0.02	15 [4–23]	21 [10–38]	0.02
Diabetes mellitus**	10/177 (1%)	5/88 (6%)	5/89 (6%)	0.99	8/90 (1%)	2/87 (2%)	0.06
**Bone data**							
DXA performed previously	29/177 (16%)	8/88 (9%)	21/89 (24%)	<0.01	25/90 (28%)	4/87 (5%)	<0.001
Osteoporosis diagnosed previously	15/177 (8%)	5/88 (6%)	10/89 (11%)	0.19	14/90 (16%)	1/87 (1%)	<0.001
Family history of osteoporosis	35/177 (20%)	18/88 (21%)	17/89 (19%)	0.76	28/90 (31%)	7/87 (8%)	<0.001
Vitamin D and/or calcium	49/177 (28%)	22/88 (25%)	27/89 (30%)	0.43	38/90 (42%)	11/87 (13%)	<0.001

Data are presented as numbers with percentage in parenthesis, normally distributed continuous variables as mean ± standard deviation and non-normally distributed variables as median with interquartile range in brackets.

One-way analysis of variance, Kruskal-Wallis' test and χ^2-test were used to test variables by groups. Risk factors, bone data and medication data were self-reported at the baseline visit. Family history of osteoporosis included first-degree relatives with known osteoporosis or a previous hip fracture.

* Only including participants who were former or active smokers. A cigarette pack year was defined as smoking one pack (containing 20 cigarettes) per day for one year.

** Diabetes mellitus including type I and II.

Abbr.: BMD, bone mineral density; DXA, dual energy X-ray absorptiometry.

Supplementary Table S3: Mean lumbar and thoracic BMD values stratified by five-year age intervals

Subject age (years)	No of subjects	Mean lumbar BMD mg/cm^3	Mean thoracic BMD mg/cm^3	Absolute ΔBMD	Relative ΔBMD
<45	10	163±34	171±33	8±17	1.06±0.12
45– <50	16	151±37	162±41	11±23	1.08±0.14
50– <55	32	135±39	147±41	12±18	1.10±0.14
55– <60	23	134±25	143±29	10±23	1.08±0.18
60– <65	46	118±34	136±32	18±17	1.18±0.19
65– <70	31	95±34	117±37	21±15	1.26±0.20
70– <75	19	93±25	114±25	22±18	1.27±0.25
31–74 (all)	177	122±38	137±37	16±19	1.16±0.20
p value*	–	<0.001	<0.001	0.06	<0.001

Data is presented as mean with ± standard deviation stratified by age grouped according to five-year age intervals. Mean lumbar BMD is the mean of L1-L3 (3LS) and mean thoracic BMD is the mean of three consecutive vertebrae (3TS) starting from the level of the left anterior descending artery.

* One-way analysis of variance was used to test overall difference between age groups.

Abbr.: BMD, bone mineral density.

Supplementary Table S4: Sex differences of bone mineral density assessed at individual vertebra

**Characteristics**	**All (n=177)**	**Women (n=90)**	**Men (n=87)**	***p value***
Mean thoracic BMD, mg/cm^3	137±37	136±42	139±32	0.61
Mean lumbar BMD, mg/cm^3	122±39	120±44	123±32	0.61
Absolute increase	16±19, p<0.001	16±18	15±19	0.97
Relative increase	1.16±0.20, p<0.001	1.17±0.20	1.15±0.19	0.50
**Mean BMD in individual vertebrae**	**All (n=177)**	**Women (n=90)**	**Men (n=87)**	**p value**
Vertebra 1	137±41	134±45	140±37	0.37
Vertebra 2	135±41	132±44	137±37	0.45
Vertebra 3	134±44	136±47	132±40	0.47
L1	125±40	125±45	125±35	0.99
L2	119±44	118±49	120±39	0.80
L3	106±47	103±48	109±46	0.44

Difference between thoracic and lumbar BMD according to sex, calculated as the absolute and relative increase. Data is presented as normally distributed continuous variables with mean ± standard deviation. Individual vertebrae are presented for the thoracic spine, vertebra 1-3 (3TS), corresponds to the three consecutive vertebrae starting at the level of the left anterior descending artery. Individual vertebrae are presented for the lumbar spine, L1-L2-L3 (3LS), corresponds to the first, second and third lumbar vertebra from the last thoracic rib-carrying vertebra.
Student's t-test was used to assess differences in BMD values between scan regions, and to test dichotomous groups.
Abbr.: BMD, bone mineral density.

Supplementary figures

Supplementary Figure S1

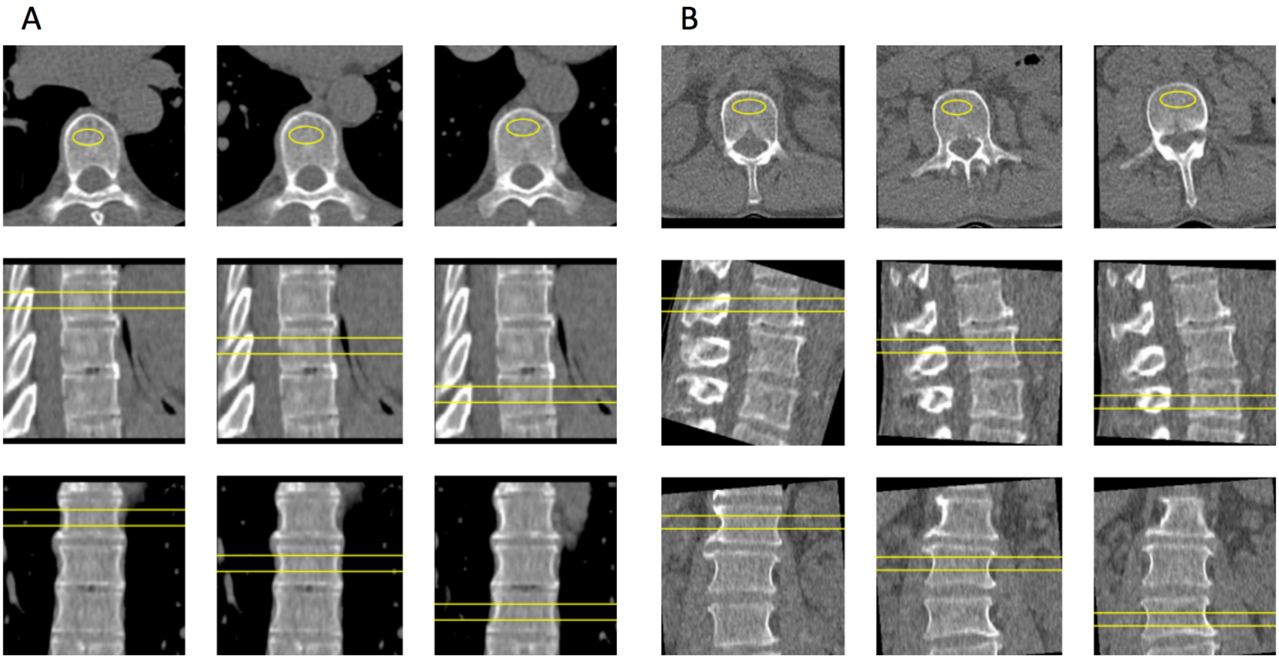

Supplementary Figure S2: From the QCT Mindways software analysis module, illustrations of (A) VOI placement in three thoracic vertebrae. (B) VOI placement in three lumbar vertebrae.

In three consecutive thoracic or lumbar vertebrae, a VOI (yellow region) was automatically placed and presented by the Mindways software analysis module in axial, sagittal and coronal view. This was followed by the possibility of manual adjustments when needed. The VOI placement and/or size was adjusted to encompass as much trabecular bone as possible while excluding cortical bone, osteophytes and the posterior venous plexus. The thoracic vertebrae (3TS) are three consecutive vertebrae starting at the level of the left anterior descending artery, corresponding to Th7 (48%), Th8 (37%), Th6 (11%), or Th9 (4%) [16]. Vertebrae in the lumbar spine (L1-L3) corresponds to three consecutive vertebrae from the last thoracic rib-carrying vertebra (3LS).

Abbr.: BMD, bone mineral density; CT, computed tomography; QCT, quantitative computed tomography; VOI, volume of interest.

Supplementary Figure S2

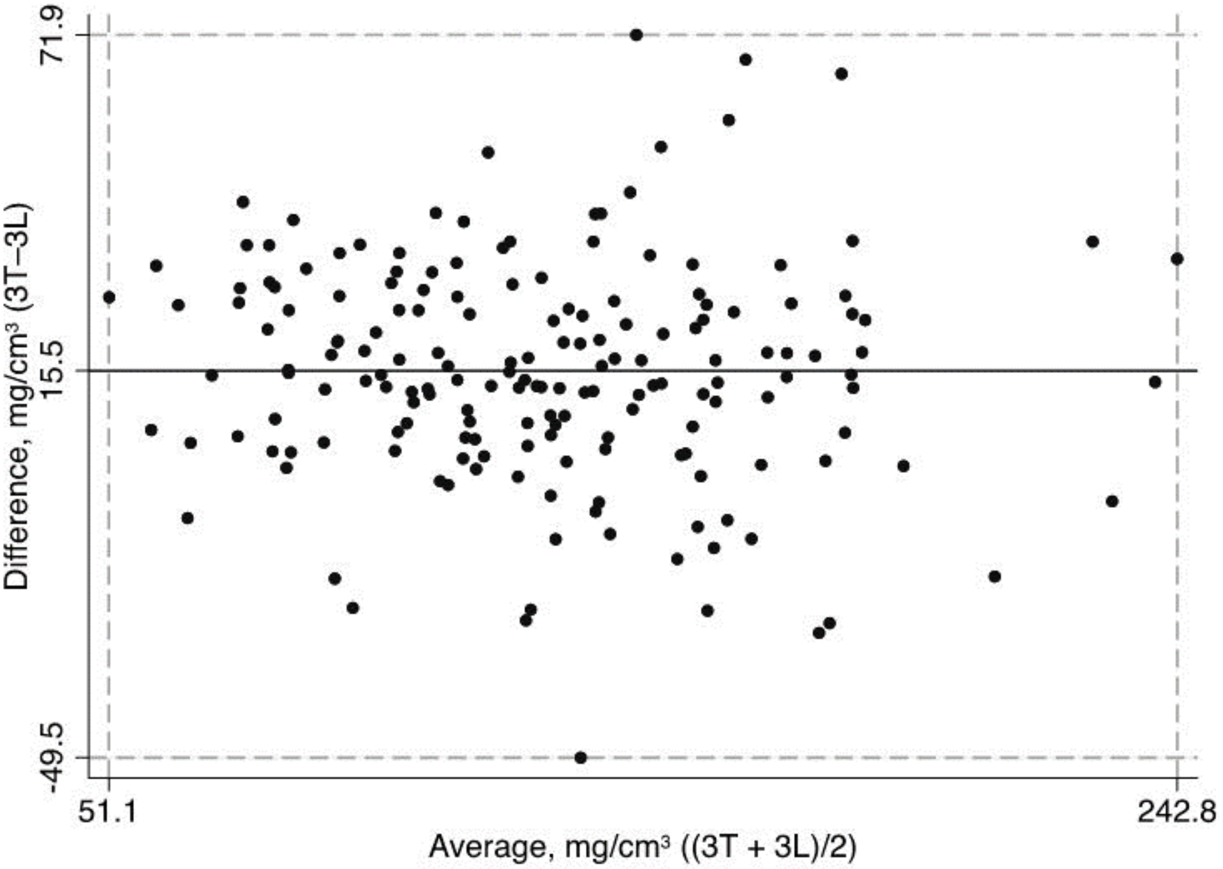

Supplementary Figure S2: Bland-Altmann plot to visualize potential systematic bias between BMD measurements performed at the thoracic and lumbar spine level with 95% prediction interval (dashed grey lines): BMD [-49.5; 71.9] mg/cm^3, mean difference: 15.5 mg/cm^3 (95%CI: 12.7–18.2) (black line). Range for average BMD [51.1; 242.8].
Abbr.: BMD, bone mineral density
